# Self-testing for HIV, HBV, and HCV using finger-stick whole-blood multiplex immunochromatographic rapid test: A pilot feasibility study in sub-Saharan Africa

**DOI:** 10.1371/journal.pone.0249701

**Published:** 2021-04-09

**Authors:** Serge Tonen-Wolyec, Roland Marini Djang’eing’a, Salomon Batina-Agasa, Charles Kayembe Tshilumba, Jérémie Muwonga Masidi, Marie-Pierre Hayette, Laurent Bélec

**Affiliations:** 1 Ecole Doctorale Régionale D’Afrique Centrale en Infectiologie Tropicale, Franceville, Gabon; 2 Faculty of Medicine and Pharmacy, University of Kisangani, Kisangani, Democratic Republic of the Congo; 3 Laboratory of Analytical Chemistry, Faculty of Medicine, University of Liège, Liège, Belgium; 4 National AIDS and STIs Reference Laboratory, Kinshasa, Democratic Republic of the Congo; 5 Faculty of Medicine, University of Kinshasa, Kinshasa, Democratic Republic of the Congo; 6 Laboratory of Microbiology, University Hospital of Liège, Faculty of Medicine, University of Liège, Liège, Belgium; 7 Laboratoire de virologie médicale et oncologique, Hôpital Européen Georges Pompidou, Assistance Publique-Hôpitaux de Paris, Paris, France; 8 Faculté de Médecine Paris Descartes, Université de Paris, Paris Sorbonne Cité, Paris, France; Centre de Recherche en Cancerologie de Lyon, FRANCE

## Abstract

**Background:**

The burden of HIV, HBV, and HCV infections remains disproportionately high in sub-Saharan Africa, with high rates of co-infections. Multiplex rapid diagnostic tests for HIV, HBV and HCV serological testing with high analytical performances may improve the “cascade of screening” and quite possibly the linkage-to-care with reduced cost. Based on our previous field experience of HIV self-testing, we herein aimed at evaluating the practicability and acceptability of a prototype finger-stick whole-blood Triplex HIV/HCV/HBsAg self-test as a simultaneous serological screening tool for HIV, HBV, and HCV in the Democratic Republic of the Congo (DRC).

**Methods:**

A cross-sectional multicentric study consisting of face-to-face, paper-based, and semi-structured questionnaires with a home-based and facility-based recruitment of untrained adult volunteers at risk of HIV, HBV, and HCV infections recruited from the general public was conducted in 2020 in urban and rural areas in the DRC. The practicability of the Triplex self-test was assessed by 3 substudies on the observation of self-test manipulation including the understanding of the instructions for use (IFU), on the interpretation of Triplex self-test results and on its acceptability.

**Results:**

A total of 251 volunteers (mean age, 28 years; range, 18–49; 154 males) were included, from urban [160 (63.7%)] and rural [91 (36.3%)] areas. Overall, 242 (96.4%) participants performed the Triplex self-test and succeeded in obtaining a valid test result with an overall usability index of 89.2%. The correct use of the Triplex self-test was higher in urban areas than rural areas (51.2% *versus* 16.5%; aOR: 6.9). The use of video IFU in addition to paper-based IFU increased the correct manipulation and interpretation of the Triplex self-test. A total of 197 (78.5%) participants correctly interpreted the Triplex self-test results, whereas 54 (21.5%) misinterpreted their results, mainly the positive test results harboring low-intensity band (30/251; 12.0%), and preferentially the HBsAg band (12/44; 27.3%). The rates of acceptability of reuse, distribution of the Triplex self-test to third parties (partner, friend, or family member), linkage to the health care facility for confirmation of results and treatment, and confidence in the self-test results were very high, especially among participants from urban areas.

**Conclusions:**

This pilot study shows evidence for the first time in sub-Saharan Africa on good practicability and high acceptability of a prototype Triplex HIV/HCV/HBsAg self-test for simultaneous diagnosis of three highly prevalent chronic viral infections, providing the rational basis of using self-test harboring four bands of interest, *i*.*e*. the control, HIV, HCV, and HBsAg bands. The relatively frequent misinterpretation of the Triplex self-test points however the necessity to improve the delivery of this prototype Triplex self-test probably in a supervised setting. Finally, these observations lay the foundations for the potential large-scale use of the Triplex self-test in populations living in sub-Saharan Africa at high risk for HIV, HBV, and HCV infections.

## Introduction

Human immunodeficiency virus (HIV), hepatitis B virus (HBV), and hepatitis C virus (HCV), are the three most common chronic viral infections all over the world sharing similar transmission routes including sexual, blood contact, and injecting drug usage [1–4]. Furthermore, HIV infection affects the natural history of HBV and HCV infections by accelerating progression to chronic liver disease due to drug-related hepatotoxicity and reactivation of hepatitis [5, 6]. Worldwide, HBV infection accounts for about 248 million chronic infections, HCV for an estimated 110 million, and HIV for about 36 million [4]. The ever-increasing burden of these infections has become a growing concern [7].

The burden of HIV, HBV, and HCV infections remains disproportionately high in sub-Saharan Africa [2–4]. Indeed, certain populations such as people who inject drugs, men who have sex with men, and people living with HIV have high-risk levels of acquiring HBV and HCV [4]. With therapeutic advances and the availability of well-codified treatments, screening for HIV, HBV, and HCV infection is currently become a crucial component of an effective response to these chronic infections [4].

Although rapid diagnostic tests (RDT) have revolutionized the detection of many infectious diseases over the past two decades, particularly HIV, HBV and HCV infections, many people infected with these viruses remain unaware of their positive status, and may therefore continue to transmit the infection to others [8]. Recently, multiplex RDTs have been developed for the simultaneous diagnosis of HIV, HBV and HCV infections [9]. The use of multiplex RDT for HIV, HBV [HBV surface antigen (HBsAg)], and HCV screening has the advantage of improving the cascade of screening, prevention strategies, and linkage-to-care at a significantly reduced cost [9–11].

The development of RDTs for HIV testing has made possible the implementation of innovative tools such as HIV self-test. HIV self-testing is an innovative strategy to make testing more accessible, confidential, and available at non-traditional venues, such as pharmacies and community venues, as well as in homes, as it offers a discreet, convenient, and empowering way to test [12–14]. HIV self-testing has demonstrated high acceptability, feasibility, and accuracy in various untrained populations in sub-Saharan Africa [15–20]. The WHO currently considers HIV self-testing to be a strategy that could revolutionize HIV testing to control HIV/AIDS by 2030 [14].

Self-testing using multiplex immunochromatographic RDT could also improve simultaneous screening of HIV, HBV, and HCV infections [21]. Multiplex self-testing for HIV, HBV, and HCV could reduce the chance of missed opportunities of testing these infections outside facility-based settings [21]. Such testing strategy could be particularly beneficial for high-risk groups who are hard to reach and could also likely increase the public health impact of self-testing [22].

Multiplex RDTs for HIV, HBV and HCV serological testing primarily designed for professional use remains limited [23]. To our knowledge, their potential use as multiplex self-tests has not been yet explored. Based on our previous field experience of HIV self-testing, we herein aimed at evaluating the practicability and acceptability of a prototype finger-stick whole-blood Triplex HIV/HCV/HBsAg self-test as a simultaneous serological screening tool for HIV, HBV, and HCV in the Democratic Republic of the Congo (DRC).

## Material and methods

### Prototype immunochromatographic rapid test for HIV, HBV and HCV self-testing

The prototype finger-stick whole-blood Triplex HIV/HCV/HBsAg self-test was adapted from the IVD-labeled, immunochromatographic Triplex HIV/HCV/HBsAg (Biosynex, Strasbourg, France) designed for professional use, by re-packaging the RDT for individual use with the addition of six components placed in a pouch containing the test cassette, diluent vial, pipette, alcohol wipe, compress, lancet and dressing. The Triplex HIV/HCV/HBsAg self-test consists of manually performed, visually interpreted, qualitative, *in vitro* lateral flow immunoassays for simultaneous detection of HIV, and HCV-specific antibodies (Ab; IgG and IgM) and HBsAg in human whole-blood (venipuncture and fingerstick), serum, or plasma. The test uses synthetic antigens (gp41, gp36) able to detect antibodies against HIV-1 or HIV-2, monoclonal antibody to HBsAg to detect HBsAg, and synthetic HCV (core, NS3, NS4 and NS5) antigens to detect antibodies against HCV. The presence of the sample is checked by the assessment of blood deposit as well as by the migration control band on the strip.

The quantity of the whole-blood needed to perform the test is 50 μL.

The analytical performances of the Triplex HIV/HCV/HBsAg (Biosynex) in professional use were previously evaluated by Robin and colleagues [9]. The Triplex RDT showed high sensitivity and specificity (ranging from 99.9% to 100.0%) in the diagnosis of HIV and replicative chronic HBV and HCV infections [9]. The price of the Triplex HIV/HCV/HBsAg (Biosynex) is around 5 US $.

**Fig 1:**
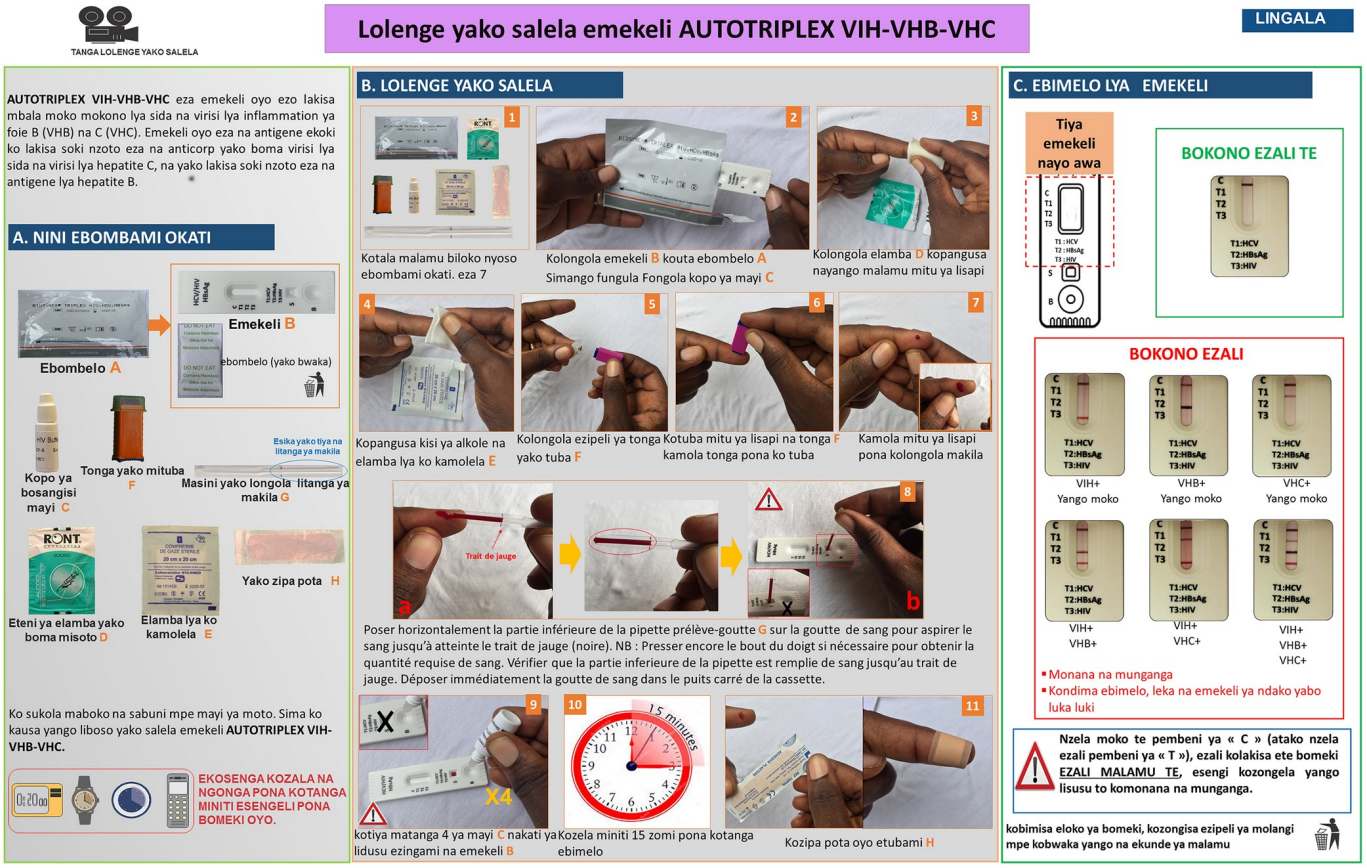
Instructions for use of the Triplex HIV/HCV/HBsAg self-test designed for the Congolese general public using typical pictures representative of the principal steps of the manufacturer’s instructions with explanations written in Lingala, which is one of the most frequently used vernacular languages of the former Province Orientale of the Democratic Republic of the Congo. Other available languages were French and Swahili. **A**. **Identification of the components**: Ⓐ Pouch, Ⓑ Test cassette, Ⓒ Diluent vial, Ⓓ Alcohol wipe, Ⓔ Compress, Ⓕ Lancet, Ⓖ Pipette, Ⓗ Dressing. **B**. **Performing the Triplex self-test**: 1. Check the contents of the kit consisting of seven components; 2. Take the self-test Ⓑ out of the bag Ⓐ and open the diluent vial Ⓒ; 3. Disinfect the chosen fingertip with the alcohol wipe Ⓓ; 4. Wipe off residual alcohol with the compress Ⓔ; 5. Remove the cap of the lancet Ⓕ; 6. Apply the lancet Ⓕ on the chosen fingertip and push the other tip to sting; 7. Press gently on the fingertip to obtain a large drop of blood; 8. Place the lower part of the pipette Ⓖ horizontally over the blood drop to aspirate the blood until the mark (black) is reached. Note: Press the fingertip again if necessary, to obtain the required amount of blood. Check that the lower part of the pipette is filled with blood up to the mark. Immediately place the drop of BLOOD into the SQUARE well of the cassette Ⓑ. 9. Shed four drops of diluent in the ROUND well DILUENT of the test cassette Ⓑ; 10. Wait exactly 15 minutes before reading the result; 11. Apply the dressing Ⓗ.

The original instructions for use (IFU) of the Triplex HIV/HCV/HBsAg (Biosynex) were adapted into a simplified but comprehensive version for the general public, with illustrations showing African people carrying out the test. The simplified IFU in French were further translated into Lingala and Swahili, which together constitute the most widely used vernacular languages in the provinces of Tshopo, Haut-Uelé, and Ituri, and printed in color A3 format. As an example, the IFU in Lingala is depicted in Fig 1. In addition, a 4-minute video on the IFU on the Triplex HIV/HCV/HBsAg self-test was edited in French language only.

All study tools, including IFUs in three languages, the demonstration video, the substudy questionnaires and the observation grids were pre-tested among 15 lay users (randomly selected at the University Hospital of Kisangani, in the DRC, among visitors of hospitalized patients) and 15 health professionals (doctors, nurses, and biologists). This pre-test consisted of assessing the comprehension of printed and video IFUs, survey questionnaires, and standardized observation forms. After integrating the remarks made by the participants in the pre-test, the tools were finally validated by the scientific management team.

Finally, ethical approval for the study was obtained from the Ethics Committee of the University of Kisangani. All ethical requirements related to the signed informed consent, anonymity, and unconditional withdrawal of participants were rigorously followed and respected.

### Study design and settings

This practicability and acceptability evaluation of the Triplex HIV/HCV/HBsAg self-test is a cross-sectional study consisting of face-to-face, paper-based, and semi-structured questionnaires with a home-based and facility-based recruitment of untrained volunteers at risk of HIV, HBV, and HCV infections.

This multicentric survey was carried out in urban (in Kisangani and Bunia, the capital city of the province of Tshopo and Ituri, respectively, and in Aru urban city of the province of Ituri) and rural [in Ariwara (rural cities of the province of Ituri), and Rungu (rural city of the province of Haut-Uelé)] areas in the DRC. The choice of these cities was justified by their high prevalences of HIV, HBV, and HCV infections and different socio-cultural and geographical contexts, as recommended by the WHO [14, 24, 25]. Concerning the facility-based recruitment, a total of 10 sites, integrating HIV, HBV, and HCV testing and care settings, were selected for the study including three sites in Kisangani (Health Centers of Neema and Sana and Kisangani Central Prison Infirmary), two sites in Bunia (Bunia Cité Hospital Center and Bunia Central Prison Infirmary), two sites in Aru (Aru General Referral Hospital and Aru Central Prison Infirmary), two site in Ariwara (Ariwara General Referral Hospital and Ariwara Central Prison Infirmary), and one site in Rungu (Rungu General Referral Hospital). Concerning the home-based recruitment, a door-to-door community approach was used to reach other key populations such as female sex workers and injection drug users.

### Study population and recruitment

**Fig 2:**
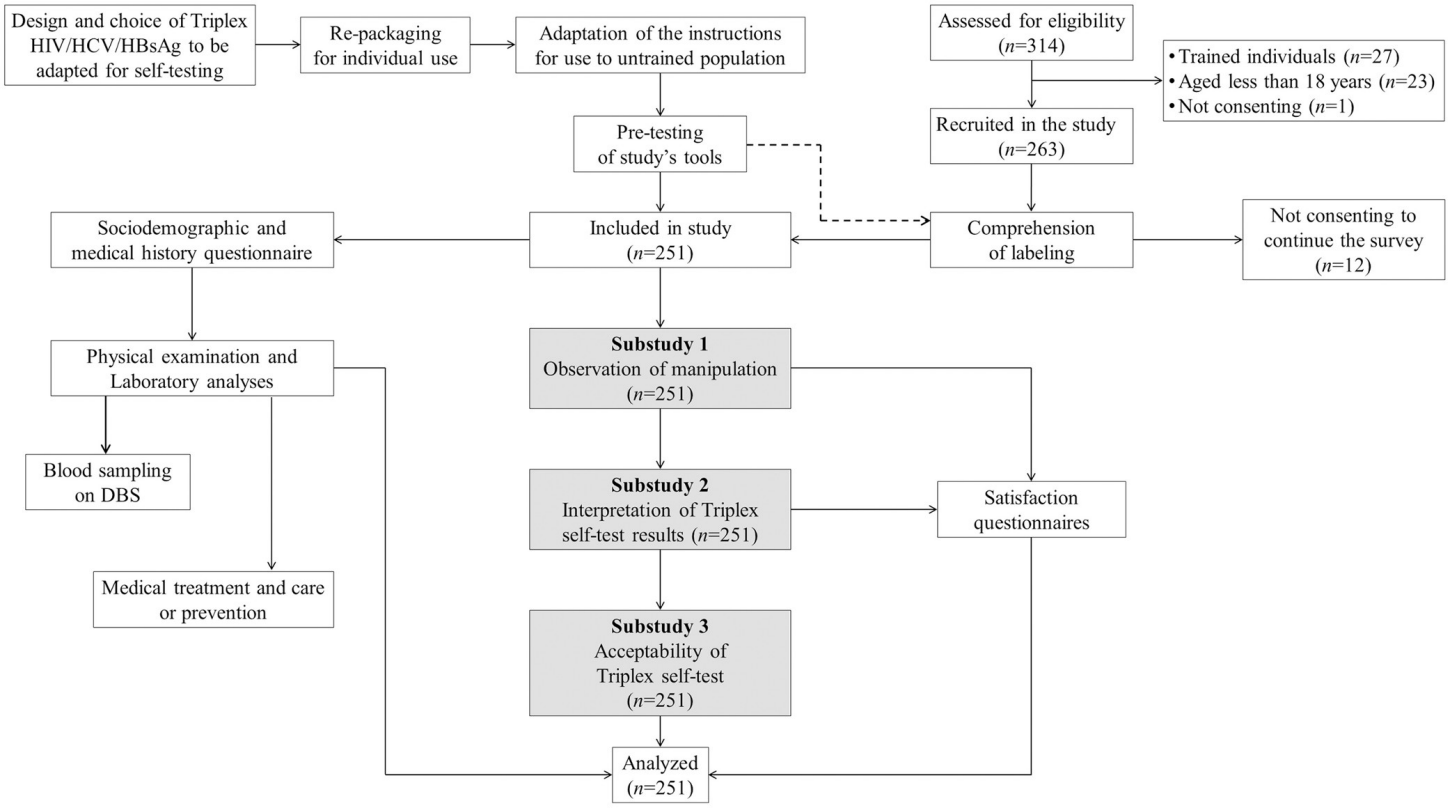
Flow chart showing the development of the Triplex HIV/HCV/HBsAg self-test, the recruitment of study participants, and their participation for each substudy. DBS: Dried blood spot.

All participants were volunteers recruited from the general public (symptomatic patients visiting health facilities because of jaundice, sexually transmitted infections, and/or long-term fever) and key populations at the study sites or home. Eligible participants were between 18 and 49 years of age, at high risk of HIV, HBV, and HCV infections, and unaware of their HIV, HBV, and HCV serostatus; they had self-professed their ability to read the IFU of the Triplex HIV/HCV/HBsAg self-test in French, Lingala, or Swahili; they agreed to undergo HIV, HBV, and HCV screenings, and gave written informed consent to participate in the study. All trained individuals (physicians, nurses, and biologists) in rapid diagnostic tests, those on antiretroviral treatment or pre-exposure prophylaxis, and those non-compliant for the study criteria, were excluded. The respondent-driven sampling method was used to recruit key populations in the community, while a consecutive inclusion was used in facilities. The recruitment process is depicted in Fig 2.

### Practicability study outcomes

The study was divided into three substudies. It was conducted by the trained health care workers so-called “observers”, based on previously acquired experience from WHO recommendations for evaluating the practicability of HIV self-tests [16, 24, 26]. A face-to-face, paper-based, and semi-structured questionnaires were used to obtain the data on the socio-demographic characteristics, medical history of study participants, participants’ opinions or levels of satisfaction about the practicability of the Triplex HIV/HCV/HBsAg self-test, and participants’ acceptability of and preferences for the Triplex self-test.

#### Substudy 1. Observation of manipulation

After signing the consent form of participation, participants were asked to read the printed instruction for use for comprehension and, if they needed to, to follow the video version instruction via the tablets managed by the research team. After reading and understanding the IFU, the participants were asked to decide whether or not to continue the Triplex HIV/HCV/HBsAg self-testing. In a private setting supervised by an observer, each participant received a box containing the Triplex HIV/HCV/HBsAg self-test. Participants were then asked to carry out the Triplex self-test by themselves in front of the observer. The observer was responsible for recording the respect or not of each step, eventual appeal for verbal assistance (mimicking telephone support), as well as any difficulties and errors on a standardized sheet. The successful performance of the Triplex HIV/HCV/HBsAg self-test was conditioned by the presence of the control band on the test strip. At the end of the session, the satisfaction of participants was evaluated using a dedicated questionnaire, including items on the experience with the Triplex HIV/HCV/HBsAg self-test, the understanding of the IFU, the recognition of the components of the HIV self-test, the sample collection and transfer, the overall performance of the Triplex self-test, and the ability to surmount the difficulties encountered.

#### Substudy 2. Reading and interpretation of Triplex self-test results

In a private setting supervised by an observer, after 15 minute-migration, the participants were asked to read and interpret their Triplex self-test results. On a standardized sheet, participants read and recorded the presence or not of a readable band (HIV, HBsAg, HCV and control) on each test strip. The participants interpreted the final result of the Triplex HIV/HCV/HBsAg self-testing as positive, negative, or invalid. Independently, observers read, interpret, and recorded the participants’ Triplex self-test results on a standardized sheet. At the end of the session, the participants were asked to fill out the satisfaction questionnaire concerning the reading of bands and the overall interpretation of the Triplex HIV/HCV/HBsAg HIV self-test results.

#### Substudy 3. Acceptability

After the practicability session, the participants were asked to fill out the acceptability questionnaire, concerning their preference to use the Triplex HIV/HCV/HBsAg self-test in a facility or community, their affirmation of wanting to reuse later and distribute the Triplex self-test, their trust in the Triplex self-test results, and their acceptance for linkage to confirmatory testing and care in case of positivity.

Finally, the participants were moved to the next room with trained staff members to complete physical examination and for blood sampling for reference laboratory examination, using national protocol for HIV, HBV, and HCV serological testing. A token for a visit to a study health facility was given to participants recruited at home to achieve this step. Re-visitations were organized for participants who did not come to the health facilities. All participants confirmed positive were treated in health care facilities, using the national protocol for the management of HIV, HBV, or HCV. Individuals who tested negative were referred to prevention management at facility.

### Statistical analysis

All data were entered into an Excel file and analyzed on SPSS 20.0 (Chicago, IL, USA). Descriptive statistics were computed using mean (standard deviation) or median (interquartile range; IQR) for normal or skewed distribution, respectively; proportions of all categorical variables were calculated for qualitative data. The usability index was defined as the mean of the correct answers for each question related to the performing of the Triplex self-test. The Wilson score bounds were used to estimate the 95% confidence intervals (CI) [27]. The Cohen’s κ coefficient estimated the concordance between the results read by participants in connection with the expected results read by the trained observers [27]. The concordance was interpreted according the Landis and Koch scale [28] as follows: < 0 as indicating no agreement, 0–0.20 as slight, 0.21–0.40 as fair, 0.41–0.60 as moderate, 0.61–0.80 as substantial, and 0.81–1 as almost perfect agreement. The Pearson’s χ^2^ test was used for comparison of the frequencies, while the Fisher’s exact test was used when the validity conditions of the latter test were not verified. Comparisons of means used the Student’s *t* test. Finally, to delineate and control possible confounders within the study variables and determine the independent predictors of the correct use of the Triplex self-test, the need for help (substudy 1), the correct interpretation of the Triplex self-test results (substudy 2), the acceptability to reuse and distribute the Triplex self-test (substudy 3), multivariable logistic regression analysis was carried out using significant variables from the bivariate analysis. The strength of statistical associations was measured by adjusted Odds ratios (aOR) and their 95% CI. The *P*-value < 0.05 was considered as statistically significant.

## Results

### Study population

A total of 314 volunteers were assessed for eligibility, of which 263 were recruited for the study and 51 people were excluded because they were deemed trained (physicians, nurses, and biologists, *n* = 27), minors (less than 18 years old, *n* = 23), and not consenting (*n* = 1) (Fig 2). After completing the reading of the Triplex self-test IFU, 251 volunteers were finally included in the study, including 160 (63.7%) from urban area [59 (23.5%) from Kisangani, 51 (20.3%) from Aru, and 50 (19.9%) from Bunia] and 91(36.3%) from rural area [50 (19.9) from Ariwara and 41 (16.3%) from Rungu].

The demographic characteristics and past medical history of the study population are depicted in Table 1. The majority of participants were aged between 18 to 29 years, male (61.4%), single (57.4%), unemployed (55%), with a relatively high educational level and generally Christians.

**Table 1:** The demographic characteristics and medical history of the 251 study participants.

Characteristics	Overall	Symptomatic patients*	Key population*
	N = 251	N = 109	N = 142
	*n* (%)	*n* (*%*)	*n* (*%*)
*Age (years)*			
18–29	158 (62.9)	67 (61.5)	91 (64.1)
30–39	64 (25.5)	22 (20.2)	42 (29.6)
40–49	29 (11,6)	20 (18.3)	9 6.3)
*Sex*			
Male	154 (61.4)	66 (60.6)	88 (62.0)
Female^#^	97 (38.6)	43 (39.4)	54 (38.0)
*Partnership and civil status*			
Single	144 (57.4)	55 (50.5)	89 (62.7)
Separated, divorced, or widowed	16 (6.4)	6 (5.5)	10 (7.0)
Married/partnered	91 (36.3)	48 (44.0)	43 (30.3)
*Occupation*			
Student	41 (16.3)	11 (10.1)	30 (21.1)
Employed	72 (28.7)	33 (30.3)	39 (27.5)
Unemployed	138 (55.0)	65 (59.6)	73 (51.4)
*Residence*			
Urban	160 (63.7)	66 (60.6)	94 (66.2)
Rural	91 (36.3)	43 (39.4)	48 (33.8)
*Educational level*			
No formal education/ attending primary school	66 (26.3)	26 (23.9)	40 (28.2)
Attending college or technical school	163 (64.9)	69 (63.3)	94 (66.2)
Attending bachelor’s or graduate degree	22 (8.8)	14 (12.8)	8 (5.6)
*Religion*			
Catholic	100 (39.8)	41 (37.6)	59 (41.5)
Protestant	93 (37.1)	49 (45.0)	44 (31.0)
Islam	13 (5.2)	3 (2.8)	10 (7.0)
Others	45 (17.9)	16 (14.7)	29 (20.4)
*Risk of HIV*, *HBV*, *or HCV acquisition*^£^			
Low risk	56 (22.3)	34 (31.2)	22 (15.5)
Moderate risk	119 (47.4)	51 (46.8)	68 (47.9)
High risk	76 (30.3)	24 (22.0)	52 (36.6)
*Previously tested for HIV*			
Never tested	157 (62.5)	75 (68.8)	82 (57.7)
Ever tested	94 (37.5)	34 (31.2)	60 (42.3)
*Previously tested for HBV*			
Never tested	235 (93.6)	100 (91.7)	135 (95.1)
Ever tested	16 (6.4)	9 (8.3)	7 (4.9)
*Previously tested for HCV*			
Never tested	242 (96.4)	104 (95.4)	138 (97.2)
Ever tested	9 (3.9)	5 (4.6)	4 (2.8)
*Previously self-tested for HIV*, *beta HCG (for pregnancy)*, *blood glucose (for diabetes mellitus)*, *etc*.			
Never self-tested	215 (85.7)	92 (84.4)	123 (86.6)
Ever self-tested	36 (14.3)	17 (15.6)	19 (13.4)

* Among symptomatic patients, 26 (23.9%) were icteric, 34 (31.2%) had sexually transmitted infections, and only 12 (11.0%) had long-term fever (≥ 21days); and among 142 key populations, 69 (48.6%) were prisoners, of whom 31 (44.9%) were men who have sex with men, 60 (42.3%) were female sex workers, and 13 (9.2%) were injection drug users;

^#^ Overall, among women, 13 (5.2%) were pregnant;

^£^ High-risk for HIV, HBV, or HCV acquisition was defined as previous history of unprotected sex with one or more partners in the past six weeks or the following high-risk exposures in the past six months: multiple (*i*.*e*. ≥2) partners, homosexual intercourse (asked to men), using sharps, having one or more blood transfusions, and having a sexually transmitted infection. Individuals were classified as “high risk” when they had two or more high risk exposures; they were classified as “low risk” if reporting none unprotected sex with one or more partners in the past six weeks, and as “moderate risk” otherwise.

HBV: Hepatitis B virus; HCG: Human chorionic gonadotrophin; HCV: Hepatitis C virus; HIV: Human immunodeficiency virus; N: Total number; n: number.

All participants were at risk for HIV, HBV, or HCV infections. The majority of them had never been tested for HIV (62.5%), HBV (93.6%), or HCV (96.4%), and had never used self-tests (85.7%) for HIV, beta-HCG (pregnancy) or blood glucose (diabetes mellitus).

Participants included 109 (43.4%) symptomatic patients from the general public and 142 (56.6%) volunteers from key populations. Among symptomatic patients, 26 (23.9%) had jaundice, 34 (31.2%) sexually transmitted infections, and 12 (11.0%) long-term fever (*i*.*e*. ≥21 days). Among participants from key populations, 60 (42.3%) were female sex workers, 13 (9.2%) were intravenous drug users, and 69 (48.6%) were prisoners, of whom 31 (44.9%) were men who have sex with men.

### Substudy 1

The substudy 1 evaluated the ability of participants to use the Triplex HIV/HCV/HBsAg self-test in a supervised setting after completing the reading of the Triplex self-test IFU. Most participants [158 (62.9%)] used only the printed IFU, whereas 93 (37.1%) participants moreover used the video IFU. The majority (56.6%) of participants used the IFU in vernacular languages (49.0% in Lingala; 7.6% in Swahili), while the remaining used the IFU in French (43.4%). Analytical results of the usability of the Triplex HIV/HCV/HBsAg self-test are shown in Table 2.

**Table 2:** Analytical results of the observation concerning the ability of the 251 study participants to correctly use each step of the Triplex HIV/HCV/HBsAg self-test autonomously or with verbal help using the printed instruction for use *versus* the combination of printed and video instructions for use (substudy 1).

Usability checklist*	Overall (N = 251)			Using the printed IFU only (N = 158)			Using the printed and video IFU (N = 93)			*P*^β^	*P*^μ^
	Observation		Need for help	Observation		Need for help	Observation		Need for help		
	Yes	No	Yes	Yes	No	Yes	Yes	No	Yes		
	[*n* (*%*)]	[*n* (*%*)]	[*n* (*%*)]	[*n* (*%*)]	[*n* (*%*)]	[*n* (*%*)]	[*n* (*%*)]	[*n* (*%*)]	[*n* (*%*)]		
1. Did the participant read the instruction for use and affirms his understanding?	242 (96.4)	9 (3.6)	0 (0)	153 (96.8)	5 (3.2)	0 (0)	89 (95.7)	4 (4.3)	0 (0)	0.730	NA
2. Did the participant easily identify the different components of the kit?	231 (92.0)	20 (8.0)	65 (25.9)	140 (88.6)	18 (11.4)	53 (33.5)	91 (97.8)	2 (2.2)	12 (12.9)	0.009	<0.001
3. Did the participant wash his hands?	151 (100)	0 (0)	0 (0)	158 (100)	0 (0)	0 (0)	93 (100)	0 (0)	0 (0)	NA	NA
4. Did the participant properly remove the test cassette from the aluminum pouch?	244 (97.2)	7 (2.8)	55 (21.9)	151 (95.6)	7 (4.4)	45 (28.5)	93 (100)	0 (0)	10 (10.8)	0.049	0.001
5. Did the participant open the diluent vial correctly?	151 (100)	0 (0)	0 (0)	158 (100)	0 (0)	0 (0)	93 (100)	0 (0)	0 (0)	NA	NA
6. Did the participant disinfect his finger correctly?	223 (88.8)	28 (11.2)	87 (34.7)	131 (82.9)	27 (17.1)	71 (44.9)	92 (98.9)	1 (1.1)	16 (17.2)	<0.001	<0.001
7. Did the participant wipe residual alcohol with the compress?	230 (91.6)	21 (8.4)	81 (32.3)	140 (88.6)	18 (11.4)	65 (41.1)	90 (96.8)	3 (3.2)	16 (7.2)	0.024	<0.001
8. Did the participant correctly remove the cap form the lancing device?	236 (94.0)	15 (6.0)	79 (31.5)	143 (90.5)	15 (9.5)	57 (36.1)	93 (100)	0 (0)	22 (23.7)	0.002	0.041
9. Did the participant have difficulty for lancing his finger?	65 (25.9)	186 (74.1)	140 (55.8)	57 (36.1)	101 (63.9)	112 (70.9)	8 (8.6)	85 (91.4)	28 (30.1)	<0.001	<0.001
10. Did the participant have difficulty forming a blood droplet?	73 (29.1)	178 (70.9)	145 (57.8)	62 (39.2)	96 (60.8)	108 (68.4)	11 (11.8)	82 (88.2)	37 (39.8)	<0.001	<0.001
11. Did the participant have difficulty using the pipette correctly until it was filled up to the blank line?	108 (43.0)	143 (57.0)	159 (63.3)	92 (58.2)	66 (41.8)	123 (77.8)	16 (17.2)	77 (82.8)	36 (38.7)	<0.001	<0.001
12. Did the participant correctly transfer and deposit the blood into the SQUARE well of the test cassette?	231 (92.0)	20 (8.0)	97 (38.6)	139 (88.0)	19 (12.0)	73 (46.2)	92 (98.9)	1 (1.1)	24 (25.8)	0.002	0.001
13. Did the participant properly shed four drops of diluent in the ROUND well of the test cassette?	234 (93.2)	17 (6.8)	93 (37.1)	142 (89.9)	16 (10.1)	71 (44.9)	92 (98.9)	1 (1.1)	22 (23.7)	0.006	0.001
14. Did the participant start the stopwatch (or other timer)?	236 (94.0)	15 (6.0)	15 (6.0)	144 (91.1)	14 (8.9)	14 (8.9)	92 (98.9)	1 (1.1)	1 (1.1)	0.012	0.101
15. Did the participant obtain an interpretable result at the end of the process despite a missed or incorrect step?^#^	242 (96.4)	9 (3.6)	NA	150 (94.9)	8 (5.1)	NA	92 (98.9)	1 (1.1)	NA	0.101	NA
*Usability index and overall need for help*, *(% [95% CI])*^£^	89.2 [84.8–92.5]		73.3 [67.5–78.4]	84.9 [78.5–89.6]		89.9 [84.2–93.7]	96.5 [90.6–98.8]		45.2 [35.5–55.3]	<0.001	<0.001
*Minute time of Triplex self-test performance*, *mean (SD)*^$^	22.3 (8.9)			24.4 (9.4)			18.8 (6.8)			<0.001	
*Correct use without difficulties*, *errors*, *and helps (n; % [95% CI])*	97; 38.6 [32.8–44.8]			26; 16.5 [11.5–23.1]			71; 76.3 [66.7–83.8]			<0.001	

* The IFU in vernacular languages (Lingala [n = 123, 49%] and Swahili [n = 19, 7.6%]) were used more often (n = 142, 56.6%) than those written in French (n = 109, 43.4%);

^β^ *P*-value comparing the observation of manipulation when participants used printed IFU only *versus* the printed combined to video IFU, using Pearson’s χ2 test or Fisher’s exact test;

^μ^ *P*-value comparing the need for verbal help when participants used printed IFU only *versus* the printed combined to video IFU, using Pearson’s χ2 test or Fisher’s exact test;

^#^ The result was considered interpretable when a control strip was readable after the migration time recommended by the manufacturer;

^£^ The usability index was defined as the mean of correct answers for each question;

^$^ Performance began since the opening of the box containing the kit of the Triplex HIV/HCV/HBsAg self-test until the migration step, and Student *t* test used for comparing the means.

CI: Confidence interval; IFU: Instructions for use; SD: Standard deviation; N: Total number; n: Number; NA: Not applicable; *P*: *P*-value.

Overall, 242 (96.4%; 95% CI: 93.3–98.1) participants performed the Triplex self-test and succeeded in obtaining a valid test result with an overall usability index of 89.2% (95% CI: 84.8–92.5). Only 97 (38.6%; 95% CI: 32.8–44.8) participants correctly used the Triplex self-test without any difficulties, errors, and help, whereas the majority (73.3%; 95% CI: 67.5–78.4) had asked for verbal help, especially when using the lancet (55.8%), forming of a sufficient blood droplet (57.8%), and using the pipette (63.3%). The mean time of Triplex self-test performance (since the opening of the box until the migration step) was 22.3±8.9 minutes.

Interestingly, the correct use (without any difficulties, errors, and help) of the Triplex HIV/HCV/HBsAg self-test was significantly higher when using printed and video IFUs (76.3%) than when only printed IFU (16.5%) (*P* < 0.001). No difference in obtaining a valid test result was found between participants who used paper-based and video IFUs *versus* printed IFU only (98.9% *versus* 94.9%; *P* = 0.101). However, the need of assistance and the meantime of Triplex self-test performance significantly decreased (45.2% *versus* 89.9% and 18.8 minutes *versus* 24.4 minutes, respectively; *P* < 0.001) when participants used video IFU. Furthermore, the usability index significantly increased (96.5% *versus* 84.9%, *P* < 0.001) among participants using the video IFU.

Furthermore, other variables such as “type of participant (symptomatic patients or key population)”, “residence (urban or rural)”, “educational level”, “risk of HIV, HBV, or HCV acquisition”, and “language used for IFU” were significantly associated with the correct use of the Triplex HIV/HCV/HBsAg self-test and the need for help in bivariate analysis. However, in multivariate analysis using logistic regression model (Table 3), only the variables “residence”, “educational level”, and “using of video IFU” were associated with the correct use of the Triplex self-test and the need for help. Indeed, the correct use of the Triplex self-test was higher among participants from urban areas than among those from rural areas (51.2% *versus* 16.5%; aOR: 6.9 [95% CI: 2.8–16.9]; *P* < 0.001). The majority of participants with high educational level used correctly the test without any difficulties, errors, and helps (68.2%; aOR: 10.1 [95% CI: 1.9–53.0]; *P* = 0.006). The use of video IFU increased the correct use of the Triplex HIV/HCV/HBsAg self-test (76.3%; aOR: 17.9 [95% CI: 8.2–39.2]; *P* < 0.001). Simultaneously, the need for help decreased among participants from urban areas by comparison with those from rural areas (64.4% *versus* 89.0%; aOR: 0.3 [95% CI: 0.1–0.6]; *P* = 0.002), among those having high educational level by comparison with those with low educational level (36.4% *versus* 86.4%; aOR: 0.08 [95% CI: 0.02–0.4]; *P* = 0.001), and among those using the video IFU in addition to printed IFU by comparison with those only using printed IFU (45.2% *versus* 89.9%; aOR: 0.1 [95% CI: 0.05–0.2]; *P* = 0.001).

**Table 3:** Multivariate regression analysis of factors associated with the correct use without difficulties, errors, and helps (substudy 1), the need for verbal help when performing the self-test (substudy 1), and the correct interpretation of the Triplex HIV/HCV/HBsAg self-test results (substudy 2) among the 251 study participants.

Characteristics	Correct use without difficulties, errors, and helps			Need for verbal help when performing the self-test (N = 184)			Correct interpretation of self-test results (N = 197)		
	Yes	aOR (95% CI)	P*	Yes	aOR (95% CI)	P*	Yes	aOR (95% CI)	P*
	[*n* (%)]			[*n* (%)]			[*n* (%)]		
*Type of participant*									
Symptomatic patients	33 (30.3)	1	1	84 (77.1)	1	1	91 (83.5)	1	1
Key population	64 (45.1)	3.3 (1.5–7.2)	0.003	100 (70.4)	0.6 (0.3–1.2)	0.133	106 (74.6)	0.5 (0.3–1.1)	0.086
*Residence*									
Rural	15 (16.5)	1	1	81 (89.0)	1	1	60 (65.9)	1	1
Urban	82 (51.2)	6.9 (2.8–16.9)	<0.001	103 (64.4)	0.3 (0.1–0.6)	0.002	137 (85.6)	3.8 (1.8–7.9)	<0.001
*Educational level*									
Low	16 (24.2)	1	1	57 (86.4)	1	1	51 (77.3)	1	1
Middle	66 (40.5)	3.4 (0.8–13.6)	0.085	119 (73.0)	0.4 (0.2–1.1)	0.058	127 (77.9)	1.1 (0.7–25.4)	0.845
High	15 (68.2)	10.1 (1.9–53.0)	0.006	8 (36.4)	0.08 (0.02–0.4)	0.001	19 (86.4)	2.6 (0.6–12.0)	0.231
*Risk of HIV*, *HBV*, *or HCV acquisition*^£^									
Low risk	19 (33.9)	1	1	42 (75.0)	1	1	47 (83.9)	1	1
Moderate risk	43 (36.1)	1.5 (0.7–3.7)	0.317	92 (77.3)	0.9 (0.3–3.2)	0.467	93 (78.2)	0.9 (0.4–1.8)	0.687
High risk	35 (46.1)	1.6 (0.6–4.5)	0.371	50 (65.8)	0.7 (0.2–1.9)	0.106	57 (75.0)	0.6 (0.2–1.5)	0.582
*Language used*									
French	61 (56.0)	3.4 (1.4–8.2)	0.006	65 (59.6)	0.5 (0.2–1.1)	0.097	87 (79.8)	1.0 (0.4–2.2)	0.920
Swahili	5 (26.3)	0.7 (1.9–2.9)	0.734	16 (84.2)	1.2 (0.3–5.0)	0.840	18 (94.7)	4.7 (0.6–39.1)	0.144
Lingala	31 (25.2)	1	1	103 (83.7)	1	1	92 (74.8)	1	1
*Using of video IFU*									
No	26 (16.5)	1	1	142 (89.9)	1	1	123 (77.8)	1	1
Yes	71 (76.3)	17.9 (8.2–39.2)	<0.001	42 (45.2)	0.1 (0.05–0.2)	<0.001	74 (79.6)	1.4 (0.7–2.8)	0.382

* *P*-value calculated using logistic regression analysis;

^μ^ Educational level was categorized according to the educational system of the Democratic Republic of the Congo, as follows: (i) low: unschooled or attending primary school; (ii) middle: attending college or technical school; and (iii) high: attending Bachelor’s or graduate degree;

^£^ High-risk for HIV, HBV, or HCV acquisition was defined as previous history of unprotected sex with one or more partners in the past six weeks or the following high-risk exposures in the past six months: multiple (*i*.*e*. ≥2) partners, homosexual intercourse (asked to men), using sharps, having one or more blood transfusions, and having a sexually transmitted infection. Individuals were classified as “high risk” when they had two or more high risk exposures; they were classified as “low risk” if reporting none unprotected sex with one or more partners in the past six weeks, and as “moderate risk” otherwise.

aOR: Adjusted Odds ratio; CI: Confidence interval; IFU: Instructions for use; N: Total number; n: Number; *P*: *P*-value.

Results of the satisfaction questionnaire concerning the overall performance of the Triplex HIV/HCV/HBsAg self-test are shown in Table 4. Most (90.9%) participants found that the IFU was easy to understand (35.5% very easy; 55.4% rather easy) without difference between the users of printed IFU and those of printed combined to video IFUs; 94% responded that the identification of the different components of the Triplex self-test was easy (37.8% very easy; 56.2% rather easy). When asked about the sample collection and the sample transfer, 24.3% and 52.6% of participants using the printed IFU only found these steps difficult, respectively, whereas only 17.2% and 21.5% of those using the printed and video IFUs found these steps difficult, respectively. Finally, 87.7% of participants responded that the overall performance of the Triplex self-test was easy (28.3% very easy; 59% rather easy).

**Table 4:** Items and analytical results of the satisfaction questionnaire concerning the overall performance of the Triplex HIV/HCV/HBsAg self-test (substudy 1) and interpretation of the Triplex self-test results (substudy 2).

Satisfaction questionnaire	Overall	Using the printed IFU only	Using the printed and video IFU	P*
	N = 251	N = 158	N = 93	
	[*n* (*%*)]	[*n* (*%*)]	[*n* (*%*)]	
How did you find the understanding of IFU of the Triplex self-test?				
Very easy	89 (35.5)	44 (27.8)	45 (48.4)	0.253
Rather easy	139 (55.4)	97 (61.4)	42 (45.2)	
Rather difficult	14 (5.6)	10 (6.3)	4 (4.3)	
Very difficult	9 (3.6)	7 (4.4)	2 (2.2)	
How did you find the identification of the different components of the Triplex self-test?				
Very easy	95 (37.8)	45 (28.5)	50 (53.8)	0.002
Rather easy	141 (56.2)	98 (62.0)	43 (46.2)	
Rather difficult	11 (4.4)	11 (7.0)	0 (0)	
Very difficult	4 (1.6)	4 (2.5)	0 (0)	
How did you find the sample collection?				
Very easy	71 (28.3)	34 (21.5)	37 (39.8)	0.044
Rather easy	119 (47.4)	79 (50.0)	40 (43.0)	
Rather difficult	55 (21.9)	40 (25.3)	15 (16.1)	
Very difficult	6 (2.4)	5 (3.2)	1 (1.1)	
How did you find the blood sample transfer?				
Very easy	30 (12.0)	11 (7.0)	19 (20.4)	<0.001
Rather easy	118 (47.0)	64 (40.5)	54 (58.1)	
Rather difficult	92 (36.7)	72 (45.6)	20 (21.5)	
Very difficult	11 (4.4)	11 (7.0)	0 (0)	
How did you find the overall performance of the Triplex self-test?				
Very easy	71 (28.3)	37 (23.4)	34 (36.6)	0.030
Rather easy	148 (59.0)	95 (60.1)	53 (57.0)	
Rather difficult	29 (11.6)	23 (14.6)	6 (6.5)	
Very difficult	3 (1.2)	3 (1.9)	0 (0)	
How did you find the reading of bands after migration?				
Very easy	83 (33.1)	43 (27.2)	40 (43.0)	0.666
Rather easy	143 (57.0)	98 (62.0)	45 (48.4)	
Rather difficult	24 (9.6)	16 (10.1)	8 (8.6)	
Very difficult	1 (0.4)	1 (0.6)	0 (0)	
How did you find the interpretation of the Triplex self-test results?				
Very easy	64 (25.5)	29 (18.4)	35 (37.6)	0.844
Rather easy	145 (57.8)	102 (64.6)	43 (46.2)	
Rather difficult	39 (15.5)	25 (15.8)	14 (15.1)	
Very difficult	3 (1.2)	2 (1.3)	1 (1.1)	
How did you find your ability to surmount the difficulties encountered?				
Very easy	42 (16.7)	15 (9.5)	27 (29.0)	0.096
Rather easy	181 (72.1)	121 (76.6)	60 (64.5)	
Rather difficult	24 (9.6)	19 (12.0)	5 (5.4)	
Very difficult	4 (1.6)	3 (1.9)	1 (1.1)	

* *P*-value calculated using Pearson’s χ2 test or Fisher’s exact test by grouping the categories “very easy” and “rather easy” into “easy” and “very difficult” and “rather difficult” into “difficult” to make a 2x2 cross.

IFU: Instructions for use; N: Total number; n: Number; *P*: *P*-value.

### Substudy 2

The substudy 2 evaluated the ability of participants to read and interpret the Triplex HIV/HCV/HBsAg self-test results. The interpretation of the self-test results read by the trained observers was considered as the reference.

**Fig 3:**
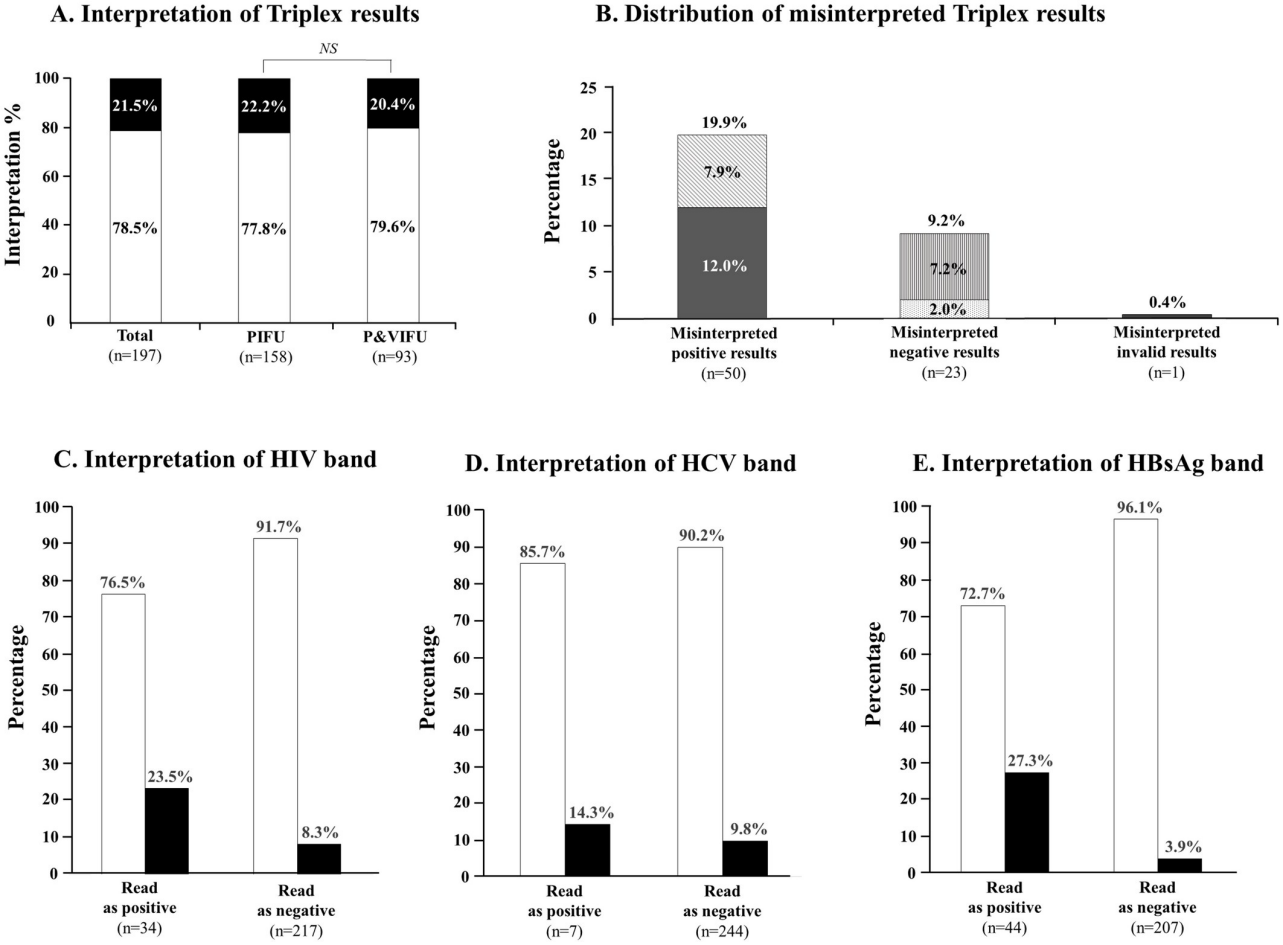
Stacked columns showing analytical results of the interpretation of the Triplex HIV/HCV/HBsAg self-test results among the 251 study participants. **A**. Overall interpretation of Triplex self-test results in the whole study population, including those using exclusively the paper-based instructions for use (PIFU) and those using both printed and video instructions for use (P&VIFU); **B**. Distribution of misinterpreted Triplex self-test results, including 50 positive tests misinterpreted as negative (dark grey; n = 30) or as positive but with confusion in band interpretation (lying grey hatches; n = 20), 23 negative tests misinterpreted as invalid (vertical grey hatches; n = 18), or positive (horizontal grey hatches; n = 5), and 1 invalid tests misinterpreted as negative (dark grey); **C**. Percentages of correctly interpreted and misinterpreted HIV band read as positive and negative; **D**. Percentages of correctly interpreted and misinterpreted HCV band read as positive and negative; **E**. Percentages of correctly interpreted and misinterpreted HBsAg band read as positive and negative. Columns in black represent misinterpretation, whereas columns in white represent correct interpretation. NS: Not significant; PIFU: Paper-based instructions for use; P&VIFU: Paper-based and video instructions for use.

The results concerning the interpretation of the Triplex self-test are depicted in Fig 3. When assessing the ability of participants to read and interpret the positive, negative, or invalid results, 197 (78.5%; 95% CI: 73.0–83.1) participants correctly interpreted the results of Triplex self-test, without statistical difference between those using printed IFU only (77.8%) and those using printed and video IFUs (79.6%), whereas 54 (21.5%; 95% CI: 16.9–27) participants misinterpreted their results (Fig 3A). Among the 54 misinterpreted Triplex test results, the majority concerned positive tests (30/54, 55.6%), including 38.0% (30/79) of the positive Triplex test results, followed by negative tests (23/54, 42.6%) corresponding to 14.1% (23/163) of the negative Triplex test results, and rarely invalid tests (1/54, 1.9%) corresponding to 11.1% (1/9) of the invalid Triplex test results. The details of the misinterpreted tests are shown in Fig 3B. Misinterpretation occurred in 30 (30/251, 12.0%; 95% CI: 8.5–16.6) positive test results with a low-intensity band which were all misinterpreted as negative and in 20 (20/251, 7.9%; 95% CI: 5.2–11.9) positive test results which were interpreted as positive but with confusion in band interpretation among participants who had difficulties to spot exactly which virus was concerned by the positivity. Misinterpretation occurred in 23 (23/251, 9.1%; 95% CI: 6.1–13.3) negative test results, including 18 (18/251, 7.2%; 95% CI: 4.6–11.1) interpreted as invalid, and 5 (5/251, 2.0%; 95% CI: 0.9–4.6) interpreted as positive. Finally, the only 1 (1/251, 0.4%; 95% CI: 0.1–2.2) misinterpreted invalid test result was interpreted as negative.

Furthermore, when evaluating the interpretation of band read as positive separately (Fig 3C–3E), the positive HBsAg band was the most frequently misinterpreted (12/44, 27.3%), followed by the positive HIV band (8/34, 23.5%) and the positive HCV band (1/7, 14.3%). Overall, the concordances between the interpretation of the HIV, HBsAg and HCV band read by participants *versus* trained observers were estimated at 0.71, 0.61 and 0.29, respectively, using Cohen’s κ coefficient calculation, yielding a substantial agreement between participants and trained observer for HIV and HBsAg bands and a fair agreement for HCV band. Finally, the overall concordance of all test bands (HIV, HBsAg, HCV) between the participants and observers was 0.59%, yielding a moderate agreement.

The interpretations of the Triplex test results by the participants and that by the observers (expected results) for the 20 positive bands concerned by the discordances are shown in Table 5. The confusion of misinterpreted positive bands concerned HIV and HBV co-infection in 15% (3/20) of cases. Overall, HIV and HBV co-infection was found among 6 participants while co-infections by HIV and HCV, HBV and HCV, or HIV, HBV, and HCV, were not found in study series.

**Table 5:** Correspondence between the interpretation of the Triplex HIV/HCV/HBsAg self-test results by the participants and by the trained observers among 20 results of the Triplex self-test correctly identified as positive but misinterpreted because of discordance of positive bands.

Number	ID	Discordance in misinterpreted positive band(s)								Concordance^β^
		Participant interpretation				Trained observer interpretation				
		Control band	HIV	HBsAg	HCV	Control band	HIV	HBsAg	HCV	
1	**#6**	+	+	–	–	+	+	+	–	**2/3**
2	**#18**	+	+	+	–	+	–	+	–	**2/3**
3	**#64**	+	+	–	–	+	–	+	–	**1/3**
4	**#83**	+	–	+	–	+	+	+	–	**2/3**
5	**#94**	+	+	–	–	+	–	–	+	**1/3**
6	**#102**	+	–	+	+	+	+	–	–	**0/3**
7	**#125**	+	+	–	+	+	–	+	–	**0/3**
8	**#130**	+	+	–	+	+	–	+	–	**0/3**
9	**#133**	+	–	+	–	+	+	+	–	**2/3**
10	**#148**	+	+	+	–	+	–	+	–	**2/3**
11	**#156**	+	+	+	+	+	–	+	–	**1/3**
12	**#162**	+	+	–	–	+	–	+	–	**1/3**
13	**#173**	+	–	+	–	+	+	–	–	**1/3**
14	**#181**	+	–	+	+	+	–	+	–	**2/3**
15	**#187**	+	+	–	+	+	+	–	–	**2/3**
16	**#198**	+	–	–	+	+	–	+	–	**1/3**
17	**#207**	+	+	–	+	+	+	–	–	**2/3**
18	**#235**	+	–	+	+	+	–	+	–	**2/3**
19	**#244**	+	+	–	+	+	–	+	–	**0/3**
20	**#249**	+	–	–	+	+	–	+	–	**1/3**

Differences are highlighted in grey.

^β^ Concordance is the n/n’ ratio corresponding to the number (n) of correct interpretation of each reactive band (compared to expected reading done by trained observers) out of the number (n’ = 3) of detectable positivity with the Triplex HIV/HCV/HBsAg self-test.

In multivariate logistic regression analysis taking account all variables associated to the correct use or need for help (substudy 1) and the correct interpretation of Triplex self-test results in bivariate analysis, only the variable “residence (urban or rural areas)” remained associated with the correct interpretation of the Triplex HIV/HCV/HBsAg self-test results (Table 3): Correct interpretation of positive tests was higher in participants from urban areas than in those from rural areas (85.6% *versus* 65.8%, aOR: 3.8 [95% CI: 1.8–7.9]; *P*<0.001).

Results of the satisfaction questionnaire concerning the interpretation of the Triplex self-test results are shown in Table 4. Most (90.1%) participants found that the reading of bands after the migration step was easy (33.1% very easy; 57.0% rather easy). When asked about the overall interpretation of Triplex self-test results, only 16.7% of participants responded that it was difficult. However, none difference concerning the reading of bands and the interpretation of the Triplex self-test results could be observed between the users of printed IFU only and those of printed combined to video IFUs.

### Substudy 3

The substudy 3 evaluated the acceptability of and preferences for the Triplex HIV/HCV/HBsAg self-test among study participants (Table 6).

**Table 6:** Analytical results of the acceptability of and the preferences for the Triplex HIV/HCV/HBsAg self-test among 251 study participants.

Characteristics	Overall	Symptomatic patients	Key populations	P*
	N = 251	N = 109	N = 142	
	*n* (%)	*n* (*%*)	*n* (*%*)	
Where would you prefer to use the Triplex HIV/HCV/HBsAg self-test?				
In facilities	39 (15.5)	17 (15.6)	22 (15.5)	0.982
In community or at home	212 (84.5)	92 (84.4)	120 (84.5)	
Would you accept to reuse the Triplex HIV/HCV/HBsAg self-test?				
No	3 (1.2)	1 (0.9)	2 (1.4)	0.760
Yes	240 (95.6)	101 (92.7)	139 (97.9)	
Not sure	8 (3.2)	7 (6.4)	1 (0.7)	
Would you accept to distribute the Triplex HIV/HCV/HBsAg self-test to your partner, friend or family member?				
No	22 (8.8)	5 (4.6)	17 (12.0)	0.059
Yes	216 (86.1)	94 (86.2)	122 (85.9)	
Not sure	13 (5.2)	10 (9.2)	3 (2.1)	
Did you trust in the results of the Triplex HIV/HCV/HBsAg self-test?				
No	7 (2.8)	2 (1.8)	5 (3.5)	0.703
Yes	230 (91.6)	95 (87.2)	135 (95.1)	
Not sure	14 (5.6)	12 (11.0)	2 (1.49	
Would you accept to go to the laboratory to confirm your result if it is positive for one or more infections?				
No	29 (11.6)	12 (11.0)	17 (12.0)	0.832
Yes	214 (85.3)	93 (85.3)	121 (85.2)	
Not sure	8 (3.2)	4 (3.7)	4 (2.8)	
Would you accept to receive treatment in case of confirmation of positivity?				
No	8 (3.2)	2 (1.8)	6 (4.2)	0.472
Yes	240 (95.6)	106 (97.2)	134 (94.4)	
Not sure	3 (1.2)	1 (0.9)	2 (1.4)	

* *P*-value calculated using Pearson’s χ2 test or Fisher’s exact test, using only "Yes" and "No" response categories, excluding therefore the "Not sure" response category to make a 2x2 cross.

Overall, 84.5% of participants preferred to use the Triplex HIV/HCV/HBsAg self-test in community or at home as part of a comprehensive package for HIV, HVB, and HCV testing; 95.6% and 86.1% of them would accept to reuse and secondarily distribute the Triplex self-test to partners, friends or family members, respectively. Furthermore, 91.6% of participants declared to have trust in the results of the Triplex self-test; in case of any positivity, 85.3% and 95.6% were favorable to go to a laboratory or healthcare facilities, for further laboratory confirmatory testing and care, respectively. For all questions concerning the acceptability of and the preference for the Triplex HIV/HCV/HBsAg self-test, no significant differences could be observed between symptomatic patients and participants from key populations. However, acceptance to reuse and distribute the Triplex self-test was significantly higher among participants using both printed and video IFUs compared to those using only the printed IFU (acceptance to reuse: 98.6% *versus* 92.9%, *P* = 0.024; acceptance to distribute: 92.4% *versus* 81.9%, *P* = 0.009), and among those from urban *versus* rural areas (acceptance to reuse: 98.5% *versus* 91.3%, *P* = 0.019; acceptance to distribute: 92.5% *versus* 81.3%, *P* = 0.007) (data not shown). Finally, in multivariate logistic regression analysis, no variables were associated with the reuse and distribution of the Triplex self-test.

## Discussion

We herein report on our recent experience of the practicability and acceptability of a prototype finger-stick whole-blood Triplex HIV/HCV/HBsAg self-test as a simultaneous serological screening RDT for HIV, HBV, and HCV infections among adult volunteers living in the DRC, evaluated according to our previous field expertise of HIV self-testing and to the WHO recommendations for the practicability assessment of self-testing tools [14, 25]. Overall, the vast majority of participants showed a good ability to perform the Triplex HIV/HCV/HBsAg self-test until obtaining a valid test result. Although practical difficulties and need for help were frequently encountered among participants who used only the printed IFU, these difficulties decreased significantly when participants used both paper-based and video IFUs. The majority of the participants correctly interpreted their Triplex self-test results as positive, negative, or invalid. However, misinterpretation of test results was relatively frequent, in about one-fifth of participants, occurring mainly with positive test results harboring low-intensity band. Furthermore, among participants who correctly interpreted their self-test results as positive, a minority were unable to accurately recognize the virus concerned by the positive band. Finally, the rates of acceptability of reuse, distribution of the Triplex self-test to third parties (partner, friend, or family member), linkage to the health care facility for confirmation of results and treatment, and confidence in the self-test results were very high in our series, especially among participants from urban areas. Taken together, this pilot study shows evidence on good practicability and high acceptability of a prototype finger-stick whole-blood Triplex HIV/HCV/HBsAg self-test for simultaneous diagnosis of three chronic viral infections of high burden in the DRC. These observations also point the risk of misinterpretation of the Triplex self-test results, at least in the study design and context. These findings provide the observational basis for the possibility of using with high confidence rapid diagnostic self-tests harboring four bands of interest, *i*.*e*. in the case of the prototype Triplex self-test, the HIV, HBsAg, HCV, and control bands. Finally, our observations lay the foundations for the potential large-scale use of the Triplex self-test in populations living in sub-Saharan Africa at high risk for HIV, HBV, and HCV infections, to improve the “cascade of screening” and quite possibly linkage-to-care with reduced cost.

### Substudy 1

The substudy 1 is the first essential step in the evaluation of a self-test to assess the ability of participants to understand the IFU and to perform the test, in a supervised environment.

The first observation is that the majority (96.4%) of participants were able to correctly or sufficiently understand the IFU in order to obtain a valid and interpretable Triplex self-test. This finding is in full agreement with several previously published field experiences in sub-Saharan Austral, Central and West Africa demonstrating the ability in lay adults to correctly perform an immunochromatographic finger-stick whole-blood self-test, as previously reported for HIV self-testing [16, 18–20, 29, 30]. In fact, it appears clearly that the Triplex self-test kit with its package insert and components is essentially identical to numerous other immunochromatographic self-test kits designed to diagnose HIV infection. As an example in a previous study in Kisangani and Bunia, DRC, we showed that 256 (79.5%) study adult participants who were not previously trained correctly used a finger-stick whole-blood HIV self-test comparable to the Triplex self-test, either autonomously or with oral assistance, and thus correctly understood the IFU, and that 317 (98.4%) of them correctly used the HIV self-test and succeeded in obtaining an interpretable result in a supervised setting [16].

The second relevant observation is that the IFU in vernacular Congolese languages were mostly chosen by just over one in two participants (56.6%). These findings emphasize the need to propose IFU in vernacular languages in addition to the classical IFU in French or English, as previously reported for HIV self-test [15, 31]. Indeed, a large number of people in Africa do not master French or English, both languages which were imported during the colonial period, which are considered in decline, especially in rural or remote areas [32].

The third important observation is that video IFU in addition to paper-based IFU significantly improved the performance of the Triplex self-test among study untrained participants. However, even with video, the use of the pipette to collect the blood droplet remained laborious, as frequently reported in sub-Saharan Africa for finger-stick whole-blood HIV self-testing during the steps of self-lancing, blood collection, and sample transfer [15, 16, 33, 34]. Nevertheless, the interest to complete the classical paper-based IFU by other instructional tools such as short video film is now clearly admitted, mainly because it increases confidence in the ability to self-test [35]. The current WHO recommendations for HIV self-testing require that all self-testers should have the possibility to access or receive assistance over the phone, through the internet, through a dedicated hotline, or with additional instructions such as video, animations, or diagrams [25]. Such recommendations could also be applied to multiplex self-testing.

The correct use of the Triplex self-test also depended on sociological variables, in particular the level of education and the fact of living in urban areas. Previous studies on HIV self-testing in sub-Saharan Africa have emphasized that low educational level may be one of the limiting factors for the practical use of self-testing tools in African cultural context [31, 36–38]. In the present study, a high educational level was significantly associated with the correct use of the Triplex self-test. In fact, the level of education could constitute a confounding factor, since in our study we found an association between a correct use of the Triplex self-test and the fact of living in urban areas. Indeed, people living in urban areas, even if they have not attended school, are basically more self-educated, because accessibility to the internet and television media, as well as to social relationships with other literate people. In any case, the WHO recommends directly assisted self-testing for people who do not have the full capacity for unassisted self-testing such as those with low education [14, 25].

Overall, the substudy 1 demonstrates that that a multiplex finger-stick whole-blood immunochromatographic self-test can be performed sufficiently correctly by an adult lay user living in the DRC, including reading and understanding the package leaflet and performing the test itself and in particular the collection and transfer of a drop of blood. Furthermore, our observations indicate that the multiplex self-testing approach could work well in urban Africa by emphasizing the use of video in addition to paper-based IFU, and by strengthening support measures for less educated people.

### Substudy 2

After showing that the Triplex self-test could be performed correctly, the substudy 2 evaluated the reading and interpretation of Triplex self-test results, by taking into account the self-test results read by the trained observers as the reference. Indeed, one of the major challenges of multiplex self-tests is to be able to be correctly read and interpreted, despite the large number of bands to read and possible results to interpret. In other terms, the lay user must be capable to interpret as many bands of interest on the test strip as there are diseases or conditions to be diagnosed, in this case for the Triplex self-test, 3 diseases, which corresponds to 4 bands including the control band. It is currently well recognized that the ability to correctly interpret the self-test results is considered as a delicate step in self-testing [17, 39]. This refers not only to the visual subjectivity related to good visual acuity (*i*.*e*. eye without illness) when reading and interpreting the test results, but also to the number of bands to read on the test strip.

In our series, the majority (78.5%) of the participants correctly interpreted their Triplex self-test results as positive, negative, or invalid. This observation is original by itself and has, to our knowledge, never been shown in sub-Saharan Africa, and probably in the rest of the world. It demonstrates, at least within the context of the limitations of this pilot study in the DRC, that multiplex self-testing by rapid test allowing the diagnosis of 3 diseases, can be possible with a high success rate. *Posteriori* our study also demonstrates the possibility for a lay adult to read and interpret 4 bands of interest on the immunochromatography strip, *i*.*e*. the three disease bands as well as the control band. The excellent practicability of immunochromatographic two-band self-tests (control and disease or condition), as for HIV [16, 29, 35, 40–42] or other conditions such as pregnancy self-tests [43], was already well demonstrated. We previously reported that a high rate (90.2%) of adult volunteers in Kisangani and Bunia, DRC, correctly read and interpreted immunochromatographic HIV self-test using similar cassette with 2 bands of interest (HIV and control bands) [16]. Recently in France, the rate of correct interpretation of immunochromatographic self-test for IgG and IgM against SARS-CoV-2 serological screening, with 3 bands of interest (IgG and IgM to SARS-CoV-2 and control bands) was very high (98.8%) among adult volunteers [44]. The reading and interpretation of self-test for 3 diseases, as in the present, has never been yet reported in Africa.

However, our pilot study points also that the reading and final interpretation of an immunochromatographic 4 bands self-test such as the Triplex self-self presents difficulties, at least under the conditions of the study or for the participating public. Indeed, misinterpretation of Triplex self-test results was relatively frequent, in about one-fifth of participants, occurring mainly (12.0%) with positive test results harboring low-intensity band as reported by the observers, and read as negative by the lay users. Other misinterpretation concerned less frequently negative bands interpreted as invalid and an invalid band interpreted as negative. Previous studies on HIV self-testing showed that misinterpretation occurred mainly with positive test results having a low-intensity band [39] or invalid results [26, 31]. Similarly, the majority (80.0%) of misinterpreted results of COVID-19 self-test for IgG and IgM against SARS-CoV-2 concerned a weak positive IgM band [44]. This difficulty in reading some weak positive bands and in the final interpretation of HIV self-test results was previously reported in lay users as well as trained-users during professional testing [45]. In addition, our study shows another possible source of confusion, which is to misinterpret the positivity of a band and to misdiagnose another disease. Thus, among participants who correctly interpreted their self-test results as positive, a minority (7.9%) were unable to accurately recognize the virus concerned by the positive band, most often the HBsAg band, then the HIV band and finally the HCV band, for an unknown reason.

All in all, our study shows that the sources of interpretation errors with the Triplex self-test are not only frequent, but also multiple, concerning all the possibilities of interpretation (positive, negative and invalid), and all the bands of interest (HIV, HBsAg, HCV and control bands). Thus, the final interpretation of the 4 bands of the Triplex self-test can be difficult for a minority of adult lay users. Taken together, the step of reading and interpreting the Triplex self-test appears to constitute the Achilles heel of this type of immunochromatographic self-test, at least for one category of participants and under the conditions of our pilot study.

Among possibly involved sociological factors, the fact of living in urban areas was associated with the correct interpretation of the Triplex self-test results, suggesting again that the level of education or the educational context could play a determining role in the ability to read and interpret a relatively complex test for a lay user, as previously reported for HIV self-testers [15–18, 31, 33, 38, 40].

The causes of misinterpretation of the Triplex self-test should be the subject of a separate study, so that it does not constitute an obstacle for a proper multiplex self-testing approach in the African context. Indeed, it is probably possible to limit the frequency of misinterpretation, by insisting more specifically in the paper-based and video IFUs on the risks of reading errors and on the need to request assistance in the slightest doubt. Nevertheless, the difficulties of reading and interpreting the Triplex self-test must be resolved at least in part so that this type of immunochromatographic self-test can be carried out on a large scale, unless this test should be restricted to use in an assisted environment, as previously reported for HIV self-testing [46].

### Substudy 3

Our results showed an overall high acceptability of the Triplex self-test, which is reminiscent to the field experiences previously reported in sub-Saharan Africa on the secondary distribution of HIV self-test kits [18, 40, 47–49]. Furthermore, the acceptance to reuse and distribute the Triplex self-test was particularly elevated, suggesting that the promotion of the Triplex self-test can be achieved *in fine* by the users themselves. This feature could be used to reach some hard-to-reach individuals such as key populations, who will receive Triplex self-test kits distributed by the members of their own social networks. This approach was reported to be highly efficient among key populations, such as people who inject drugs, men who have sex with men, and prisoners for supporting the index testing strategy [18, 47, 49, 50]. Although our study demonstrated a high acceptance rate for secondary distribution of Triplex self-test kits, there is obviously a need for qualitative research to clarify this issue. Ultimately, the acceptability of rapid, multiplex self-tests in sub-Saharan Africa will depend on the concerned groups of population (key populations, symptomatic patients, voluntary blood donors, etc.), their usefulness in clinical decision-making, their analytical performances and cost, and their ability to be integrated into the health care systems [51].

### Strengths and limitations

To our knowledge, this is the first study is a French-speaking country in Africa to assess the practicability and acceptability of the Triplex self-test, as a novel approach for rapid, multiple and self-made screening for HIV, HBV, and HCV. Furthermore, our observations demonstrate the possibility of correctly interpreting four bands on the strip of a rapid diagnostic test by lay users from general adult population and key populations living in sub-Saharan Africa. Taken together, the study reinforces the interest of self-testing of infectious diseases in Africa. However, the study has some limitations. Thus, the study takes place in a Congolese socio-cultural context, and does not address the human diversity in sub-Saharan Africa, whose practicability and acceptability of self-testing could be different and specific according to the groups of populations. From this point of view, our study only included adults, while adolescents constitute target population for HIV self-testing [52–54]. Finally, our study did not assess the analytical virological performances of Triplex in the DRC.

### Conclusions and perspectives

HIV, HBV and HCV infections are endemic in Africa, with frequent HIV-HBV and HIV-HCV co-infections [2, 3, 55, 56]. Furthermore, prevention of mother-to-child transmission of HBV is a priority in sub-Saharan Africa [56], and adapted screening strategies for HBV infection as well HIV infection in pregnant women are needed [57]. As twin epidemics in key populations, combination integrated multi-disease assays that allow for multiplex testing of HIV, HBV and HCV infections using a single sample would improve the efficiency of screening programs and outcomes in linkage-to-care [2, 8, 10, 51, 58].

Among numerous diagnostic tools and strategies for the screening of HIV, HBV and HCV infections in Africa, remote self-testing outside clinic settings and with the person themselves implementing the test may constitute a potential innovation to enhance testing access and coverage [25, 59, 60]. Research literature on HIV self-testing indicates benefits in terms of convenience, privacy and managing stigma, but also highlights possible concerns around feasibility, acceptability and potential for harm [15, 19, 25, 40, 61]. Furthermore, interest in remote self-testing for HBV and HCV has been recently emphasized [59, 60, 62]. Transferability of the evidence and relevance of self-testing for HBV/HCV from the HIV field may contribute to this process. However, the experience with hepatitis self-testing is currently very limited. Internet enables an unprecedented opportunity to access online over the counter a broad range of self-tests, including rapid tests for HBV and HCV, which can be conducted by lay consumers without the help of a health professional [63]. As an example, results from a representative survey in Germany showed diagnostic self-testing for HBV and HCV in 1.4% by lay consumers on internet [64]. However, while self-testing offers a confidential testing solution for customers, a standard approach will be needed to ensure that people could have accessible pre- and post-counselling, for example by phone or internet or in an assisted community environment, as well as pathways of linkage-to-care.

Successful field experience of oral self-testing has been reported in Changchun, China [65]. Recently, Kimble and colleagues evaluated in Los Angeles, California, USA, the performance of the OraQuick HCV assay with self-collected oral fluid and found that the assay showed good performance on self-testing only when the results were interpreted by trained staff [66]. These latter reports show that oral self-testing for HCV is user-friendly to be potentially used for self-testing by untrained users. However, in London, UK, remote HCV self-testing was acceptable to some people who use drugs, although tensions with lived experience of drug use and health system access limited its relevance [67]. In any case, self-testing for HCV could be useful in a universal HCV screening program in primary and home care.

In conclusion, in the current medico-scientific context of the joint interest of multiplex rapid testing and self-testing, the preliminary results of our pilot study take on their full significance and promising relevance. The prototype finger-stick whole-blood Triplex HIV/HCV/HBsAg self-test can provide a rapid, inexpensive, equipment-free and user-friendly test for detecting HIV, HBV and HCV infections. Therefore, it could be particularly suitable for enhance the screening of three endemic viral infections largely linked in sub-Saharan Africa. Such possibilities could expand screening efforts, reducing the future burden of diseases related to unidentified HIV, HBV or HCV-infected patients and supporting attempts to care these infections and even to eliminate HCV infection. It remains however unclear whether HBV/HCV self-testing might play a similar role as previously shown for HIV infection in promoting uptake of viral hepatitis testing among high-risk African populations. The acceptability, utility and cost-effectiveness of HBV/HCV self-testing remain to be established in Africa, while linkage-to-care after screening should be likely enhanced. Finally, solving the problem of the relative complexity of the Triplex self-test in terms of reading and interpretation constitutes an important issue to address before generalizing its use. Additional support tools need to be assessed to improve the interpretation of the Triplex self-test results when using unassisted approach, while directly assisted self-testing in facilities or communities may probably increase its usability.

## Supporting information

S1 Appendix Study raw data. (XLSX)
